# Prevalence, knowledge and education level associated with secondhand smoke exposure among never-smoking women in Inner Mongolia, Northern China

**DOI:** 10.18332/tid/119162

**Published:** 2020-04-24

**Authors:** Xi Nan, Haiwen Lu, Jing Wu, Mingming Xue, Weidong Guo, Xuemei Wang

**Affiliations:** 1 Department of Health Statistics, School of Public Health, Inner Mongolia Medical University, Hohhot, China; 2 Department of Medical Imaging, Affiliated Hospital of Inner Mongolia Medical University, Inner Mongolia Medical University, Hohhot, China; 3 National Center for Chronic and Noncommunicable Disease Control and Prevention, Chinese Center for Disease Control and Prevention, Beijing, China; 4 School of Basic Medical Science, Inner Mongolia Medical University, Hohhot, China; 5 Department of Disease Prevention and Control, Health Commission of Inner Mongolia Autonomous Region, Hohhot, China; 6 Department of Nutrition and Food Hygiene, School of Public Health, Peking University Health Science Center, Beijing, China; * Contributed equally

**Keywords:** secondhand smoke, young women, knowledge, education level

## Abstract

**INTRODUCTION:**

Currently, 40.7% of women in China are exposed daily to secondhand smoke (SHS); however, research on SHS exposure among women in Inner Mongolia is limited. We aimed to investigate the prevalence of SHS exposure, knowledge about the harms of smoking and SHS, and the association between sociodemographic factors and SHS exposure among never-smoking women in Inner Mongolia, Northern China.

**METHODS:**

This study was based on a survey of chronic disease and nutrition conducted among Chinese adults in Inner Mongolia during 2015, a cross-sectional study with multi-stage stratified cluster sampling. A total 2293 never-smoking women aged ≥18 years were included in the study. Face-to-face interviews were used to collect data of sociodemographic characteristics, SHS exposure, and related knowledge. Questionnaires were administered by trained investigators, and the reliability and validity of the questionnaires were high. We performed descriptive analysis and logistic regression. All analyses were performed by IBM SPSS Statistics version 19.0.

**RESULTS:**

Of the total, 69% of young women in Inner Mongolia in 2015 reported that they were exposed to SHS, the highest rate in comparison with older and middle-aged women. A total 49.90% of young women reported being exposed to SHS every day. Respondents had insufficient knowledge regarding smoking and SHS exposure. Compared with older never-smoking women, their young counterparts had a higher risk of SHS exposure, with an odds ratio of 2.143 (95% CI: 1.647–2.787). Education level and ethnicity were also significantly associated with SHS exposure.

**CONCLUSIONS:**

This study showed that the highest rates of SHS exposure were among young never-smoking women in Inner Mongolia, and women with high education levels were less likely to be exposed to SHS. Improved public health information is needed that prioritizes SHS exposure among young women in Inner Mongolia. Health education regarding SHS exposure should be widely implemented throughout communities in this region.

## INTRODUCTION

Secondhand smoke (SHS) has become a serious public health challenge across the world. Globally, it is estimated that one-third of the population is frequently exposed to SHS, with 0.6 million individuals dying each year from SHS exposure^1^. According to the 2010 Global Adults Tobacco Survey (GATS), China as the world’s largest producer and consumer of tobacco has about 301 million current smokers, while 72.4% of non-smokers are exposed to SHS^2^. The results of previous studies show a close relationship between SHS and many factors. Socioeconomically disadvantaged households in terms of education and wealth index are the most vulnerable owing to a strong association with SHS exposure^3^. Those who live in socioeconomically disadvantaged areas and have poor education levels are usually less aware of the harm of SHS and may continue to be exposed to SHS from people around them in their daily social life^4^. The extend of SHS exposure is important, especially for young people who tend to participate in many recreational activities that are likely associated with a risk of SHS exposure^5^.

SHS exposure is more serious among poor and disadvantaged groups, especially women, who are more vulnerable to the effects of SHS than other populations. Little attention has been paid to young women as a group. Descriptions of tobacco use have typically been limited to males and have overlooked female tobacco use, in general. According to a national survey conducted in 2010, 71.6% of non-smoking women aged ≥15 years are exposed to SHS in China, and 40.7% of women are frequently exposed to SHS^6^. Nearly two-thirds of women are affected by family members who smoke, warranting greater attention^7^. One cross-sectional study in China indicated that young women were at higher risk of SHS exposure owing to the impact of their work environment, where more than 15% of women tend to start smoking^5^. The high rates of exposure to SHS are related to poor awareness about the harms of SHS. Compared with other low- or middle-income countries, adults in China have lower levels of knowledge about the hazards of smoking and SHS, and awareness is substantially lower for most of the many diseases specifically caused by SHS. Especially in China, women have less knowledge about the risks of SHS than men^8^.

SHS exposure leads to considerably more negative health consequences. Exposure to SHS increases the risk of cancer, cardiovascular disease, and respiratory problems among non-smokers. Women are more sensitive to SHS exposure, which is an important trigger for breast and cervical cancers and is associated with increased mortality in women^2^. A meta-analysis including cohort and case-control studies suggested that the risk of cervical cancer among women exposed to SHS was 1.70 times greater than in those with no SHS exposure (OR=1.70; 95% CI: 1.40–2.07)^9^. Currently, there is evidence that women in China and Korea who have been exposed to SHS have an increased risk of developing cervical cancer, with the trend in cervical cancer deaths more evident among younger women, which may have a profound effect on future societies^10,11^. Additionally, exposure to SHS during pregnancy is more likely to lead to poor health outcomes, such as low birthweight, premature birth, and neonatal congenital malformations^2^.

The prevalence of smoking in Inner Mongolia is 30.54%, which is higher than in other provinces of China^12^. As part of an unhealthy lifestyle, smoking is considered a significant risk factor for disease among women. The highest risk of lung cancer among women in Inner Mongolia is associated with smoking, and the high incidence of lung cancer is closely related to the high rate of smoking^13^. However, SHS exposure is not usually considered a direct risk factor related to disease and has been largely ignored among women in Inner Mongolia. In particular, young women in this region have poor health awareness and are more vulnerable to SHS exposure^14^. Research on the association between SHS exposure and disease is also limited. Although national smoking bans exist in China, smoke-free areas do not cover all indoor areas^15^. The Inner Mongolia Autonomous Region is located in a remote, undeveloped area with low socioeconomic and education levels among the population. Although there has been some promotion of public health information regarding the dangers of smoking, this has not led to implementation of compulsory smoke-free legislation in public places of Inner Mongolia. This suggests that more considerable attention is needed to raise awareness among women, especially young women, to decrease their exposure to SHS. Exploring the risk factors of SHS exposure among women will assist in developing targeted interventions.

To provide additional evidence in support of implementing public health strategies in Inner Mongolia directed towards minimizing exposure to SHS, we aimed to investigate among never-smoking women: 1) prevalence of SHS exposure; 2) knowledge related to the harms of smoking and SHS; and 3) sociodemographic factors contributing to SHS exposure.

## METHODS

### Study design

The data for this cross-sectional study were derived from a survey of chronic disease and nutrition among Chinese adults in Inner Mongolia conducted in 2015. The survey was organized by the Chinese Center for Disease Control and Prevention (CDC), as a major national public health service project in China. The survey was conducted across eight monitoring sites in Inner Mongolia. Multi-stage stratified cluster sampling was used to select participants aged ≥18 years. The first stratum consisted of 24 Sumu (a type of country-level administrative division in Inner Mongolia) or subdistrict offices in eight monitoring sites of Inner Mongolia; the second stratum included divisions of 48 Gacha (village) or neighborhood committees according to Sumu or subdistrict offices. Then, 45 households were chosen from each Gacha or neighborhood committee. A total of 5020 residents of Inner Mongolia (2160 households) were enrolled in the survey, including 2714 female and 2306 male respondents. This study was conducted according to the guidelines of the Helsinki Declaration 1975. All procedures involving human subjects were approved by the Ethics Committee of the National Institute for Chinese Center for Disease Control and Prevention. There were no treatments, blood drawings, or other interventions that could have an effect on the health of participants. All information given was treated as confidential and used for research purposes only. All participants provided their written informed consent.

**Figure 1:**
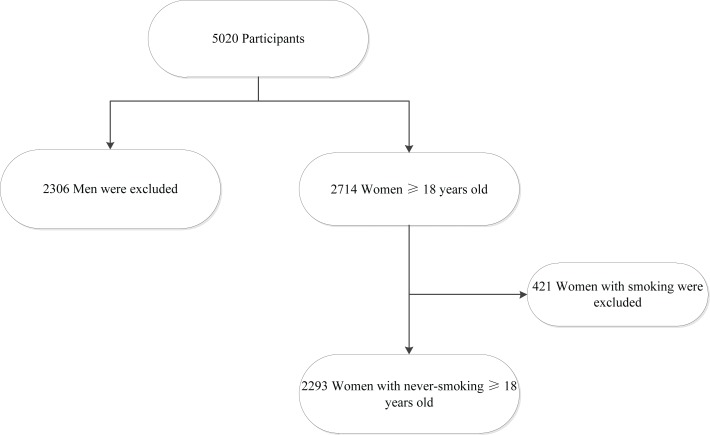
Flow diagram of detailed progress of participants selection in study on SHS exposure in Inner Mongolia, 2015

Only the survey data of women aged ≥18 years who had never smoked (participants who answered ‘no’ to the question ‘Do you currently smoke tobacco?’) were used in the analysis, and 421 female smokers were excluded. Finally, 2293 never-smoking women were included in the analysis. The detailed process of participant selection is shown in Figure 1.

### Measures

#### Exposure to SHS

The study instrument was a questionnaire from the Chinese Center for Disease Control and Prevention, which was developed by professionals and evaluated by experts; the reliability and validity of the questionnaires were high. Face-to-face interviews were used to collect information on sociodemographic characteristics, SHS exposure, and related knowledge; the interviews were conducted by trained investigators. Detailed information regarding the survey was explained to participants. Self-reported exposure to SHS was the outcome variable and was measured in this study with the following item: ‘How many days are you exposed to SHS per week?’. Participants could respond with 0, 1–3, 4–6 days or every day. We defined ‘exposed to SHS’ as self-reported SHS exposure to at least 1 day in the past 7 days, and ‘not exposed to SHS’ as no self-reported SHS exposure in 1 week. To ensure the quality of the questionnaire, the investigator reviewed all responses. If three or more responses were inconsistent with the questions, the questionnaire was re-administered to the respondent.

#### Knowledge about diseases caused by smoking and SHS exposure

Knowledge about the harmful effects of smoking was assessed with the question ‘Does smoking cause the following illness: stroke, heart disease, lung cancer?’. In addition, knowledge of the harmful effects of SHS exposure was measured with the question ‘Does SHS exposure cause the following: heart disease in adults, lung disease in children, lung cancer in adults?’. Respondents were asked to rate the degree to which they agreed with each statement (agree, disagree or uncertain); ‘agree’ was considered the correct answer, and defined as having knowledge related to smoking and SHS exposure. An additional indicator of knowledge was whether the participant agreed that low-tar cigarettes were less harmful than regular cigarettes (agree=1, disagree=2, unknown=3); a response of ‘disagree’ was defined as the correct answer.

### Definition of variables and categories

Exposure to SHS was the dependent variable. The independent variables were age group, ethnicity, marital status, education level, occupation, and residential area. Age group was categorized as follows: young women (age 15–44 years), middle-aged women (age 45–59 years), and older women (age ≥60 years). Ethnicity was categorized as Han, Mongolian or other ethnic minorities. Marital status was categorized as single, married or divorced/widowed. Education was categorized into three levels: low (illiterate), middle (primary and junior high school), and high (senior high school and above). Occupation was defined as unemployed, employed or retired. Residential area was categorized into urban or rural. The variables and their codes are shown in Table 1.

**Table 1:** Variables and coding in the analysis for never-smoking women in Inner Mongolia, 2015

*Variables*	*Categories*
Exposure to SHS	No exposure to SHS=0, exposure to SHS=1
Age	Older women=0, middle-aged women=1, young women=2
Ethnicity	Han=0, Mongolian=1, Other ethnicity=2
Marital status	Single=0, married=1, widowed/divorced=2
Education level	Low (illiterate)=0, middle (junior school)=1, high (senior high school and above)=2
Occupation	Unemployed=0, employed=1, retired=2
Residential area	Urban=0, rural=1

SHS: secondhand smoke.

### Statistical analysis

We conducted descriptive statistics for characteristics, frequency of SHS exposure, and knowledge related to low-tar cigarettes. The prevalence rates of knowledge regarding smoking and SHS (with 95% CIs) were calculated. Differences in characteristics and knowledge between the exposed and non-exposed groups or between young, middle-aged, and older women were assessed using chi-squared tests. Independent variables as predictors were included in logistic regression, comprising age group, ethnicity, marital status, education level, occupation, and residential area. Logistics regression was used to measure the association between the independent variables and exposure to SHS, and exposure to SHS was the dependent variable (no exposure to SHS = 0, exposure to SHS = 1) included in logistic regression. With α=0·05 as the significance level, p<0.05 was considered statistically significant. All statistical analyses were conducted using IBM SPSS Statistics version 19.0 (IBM Corp, Armonk, NY, USA).

## RESULTS

### Characteristics of never-smoking women in Inner Mongolia

The survey included 2714 women, and 421 who self-reported smoking were excluded. Finally, a total of 2293 never-smoking women were included in the study, among which the rate of exposure to SHS was 64.20%. Detailed comparisons of the characteristics between exposure and no exposure to SHS among participants are shown in the Supplementary Table S1. Among the total sample, the rates of exposure to SHS were the highest among young women (69.0%), followed by middle-aged women (66.5%); rates among older women were the lowest (54.1%). Most women reported being exposed to SHS, with a higher proportion among Han, married, and employed women (p<0.05) (Supplementary Table S1).

### Frequency of exposure to SHS among never-smoking women

Nearly half of young women (n=350; 49.1%) reported being exposed to SHS every day per week; 31.0% of young women reported that they were not exposed to SHS. The remaining young women reported exposure to SHS for 1–3 and 4–6 days per week (16.1% and 3.8%, respectively). The proportion of middle-aged women who were not exposed to SHS was 33.5%, and the proportions exposed to SHS for 1–3 and 4–6 days a week were 12.2% and 3.3%, respectively. In contrast, most of the older women reported no exposure to SHS (Table 2).

**Table 2:** Frequency of weekly exposure to SHS among never-smoking women in Inner Mongolia, 2015 (N=2293)

*Categories*	*Frequency of exposure to SHS per week*				*χ*^2^	*p*

	*None*	*1–3 days*	*4–6 days*	*Every day*		
Young women	221 (31.00)	115 (16.13)	27 (3.79)	350 (49.09)		
Middle-aged women	342 (33.53)	124 (12.16)	34 (3.33)	520 (50.98)	39.917	<0.001*
Elderly women	257 (45.89)	68 (12.14)	15 (2.68)	220 (39.29)		

SHS: secondhand smoke.

* Significant at p<0.001.

### Knowledge of harm caused by smoking and SHS among never-smoking women

**Figure 2:**
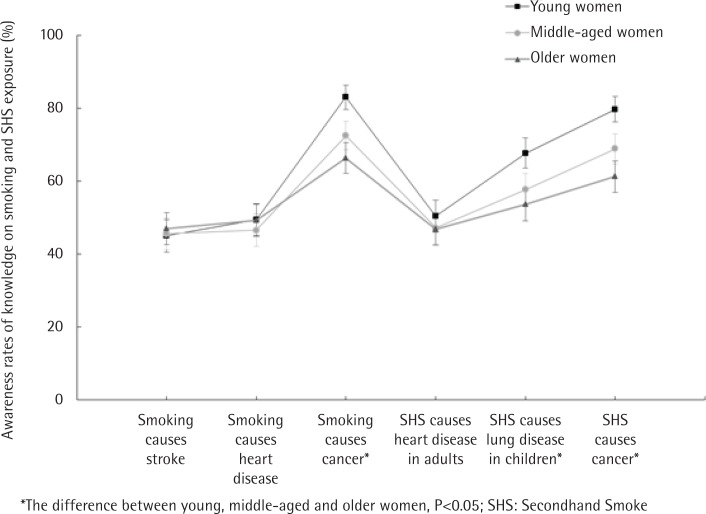
Difference of knowledge on harm caused by smoking and between older, middle-aged, and young women with never-smoking in Inner Mongolia, 2015 (N=2293)

Considering the response ‘agree’ as the correct answer regarding smoking and SHS knowledge, 45% of young never-smoking women knew that smoking could cause stroke; almost half (49.5%) knew that smokers were more likely to develop heart disease; and 83% knew that smokers were more likely to develop lung cancer. The percentages of correct answers to the question of whether SHS causes heart disease in adults, lung disease in children, and lung cancer, were 50.4%, 67.7%, and 79.7%, respectively. The proportions of middle-aged female respondents who agreed that smoking causes stroke, heart disease, and lung cancer, were 45.6%, 46.6%, and 72.5%, respectively. The proportions of respondents who agreed that SHS causes heart disease in adults, lung disease in children, and lung cancer, were 47.2%, 57.7%, and 68.9%, respectively. Few older women agreed that smoking and SHS exposure had an effect on health. The rates of respondents’ agreement with the statements that smoking causes lung cancer, and SHS causes lung disease in children, and lung cancer, were significantly different between young, middle-aged, and older women (p<0.05) (Figure 2).

### Knowledge of harm caused by smoking and SHS among young never-smoking women by exposure to SHS

**Figure 3:**
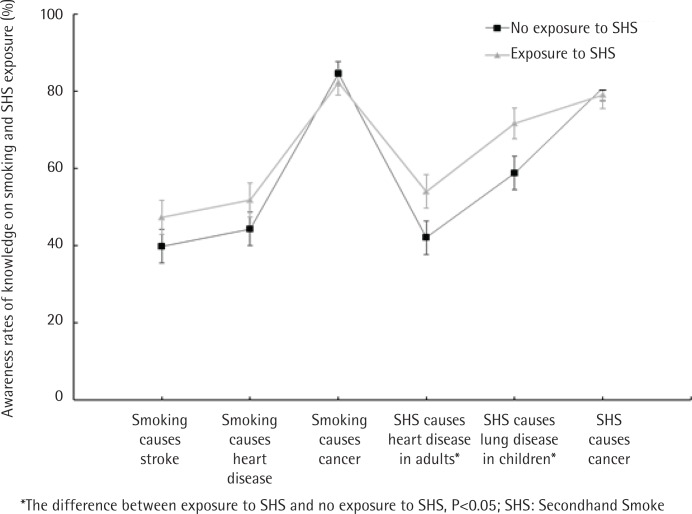
Difference of knowledge on harm caused by smoking and SHS among young never-smoking women by exposure to SHS in Inner Mongolia, 2015 (N=713)

Figure 3 shows that there were differences in the rates of agreement that SHS causes heart disease in adults and lung disease in children between young never-smoking women with and without exposure to SHS. Regarding SHS causing heart disease in adults, the exposed group had higher rates of agreement than the non-exposed group (54.1% vs 42.1%). Regarding SHS causing lung disease in children, the exposed group also had higher rates of agreement than the non-exposed group (71.8% vs 58.8%).

### Knowledge about harm of low-tar cigarettes among never-smoking women

Supplementary Table S2 shows knowledge regarding low-tar cigarettes among never-smoking women. Most young women (35.9%) agreed that low-tar cigarettes were less harmful than regular cigarettes, and 48.0% of young women were unclear whether low-tar cigarettes were less harmful than regular cigarettes. Most middle-aged women agreed that low-tar cigarettes were less harmful than regular cigarettes (30.2%). The rate of agreement that low-tar cigarettes were less harmful than regular cigarettes was 25.7% among older women. Statistically significant differences were observed between young, middle-aged, and older women, regarding the harm of low-tar cigarettes (p<0.05).

### Knowledge of harm of low-tar cigarettes among young never-smoking women by exposure to SHS

Among young never-smoking women, 38.6% of those exposed to SHS agreed that low-tar cigarettes were less harmful than regular cigarettes; these rates were significantly higher than those in the non-exposed group (29.9%). Among young women exposed to SHS, 16.5% disagreed that low-tar cigarettes were less harmful than normal cigarettes; 44.9% of these young women were unclear regarding the harm of low-tar cigarettes.

In the non-exposed group, 15.4% of young women were aware that low-tar cigarettes were less harmful than normal cigarettes, and 54.8% were unclear about the harm of low-tar cigarettes. The difference between exposed and non-exposed young women with respect to knowledge of the harm of low-tar cigarettes was statistically significant (p<0.05) (Supplementary Table S3).

### Multivariate analysis of risk factors for exposure to SHS for never-smoking women

**Figure 4:**
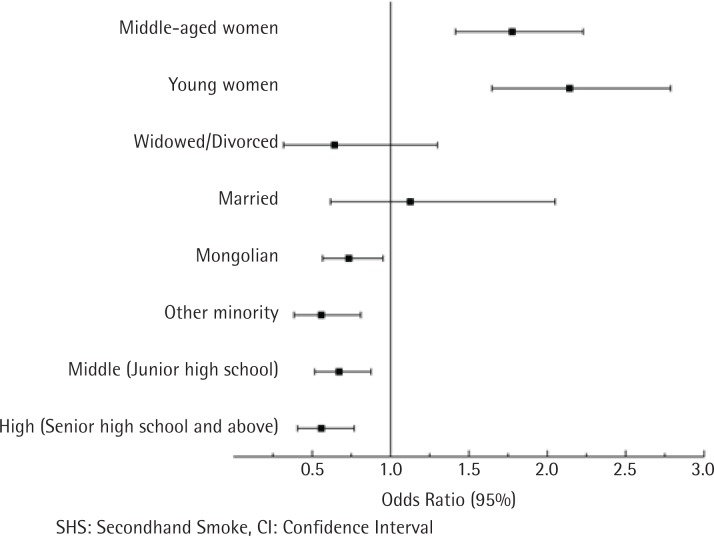
Multivariable logistic regression factors influencing SHS exposure among never-smoking women, in Inner Mongolia, 2015 (N=2293)

Multivariate analysis indicated that age group and education level were independently associated with SHS (Table 3 and Figure 4). Compared with older women, middle-aged women had a higher risk of SHS exposure (OR=1.78; 95% CI: 1.15–2.23). In particular, young women had the highest risk of SHS exposure compared with older women (OR=2.14; 95% CI: 1.65–2.79). Women completing junior school and senior high school or above were also less likely to be exposed to SHS (OR=0.67; 95% CI: 0.52–0.88 and OR=0.56; 95% CI: 0.41–0.77; respectively). Mongolian women and those of other ethnicities were less likely to be exposed to SHS than Han women.

**Table 3:** Multivariable logistic regression factors influencing SHS exposure among never-smoking women in Inner Mongolia, 2015 (N=2293)

*Factors*	*Participants*	*OR (95 % CI)*
**Age group**		
Elderly women	560	Ref.
Middle-aged women	1020	1.776 (1.415–2.229)*
Young women	713	2.143 (1.647–2.787)*
**Marital status**		
Single	51	Ref.
Married	2111	1.126 (0.618–2.050)
Widowed/divorced	131	0.643 (0.318–1.299)
**Ethnicity**		
Han	1867	Ref.
Mongolian	299	0.734 (0.567–0.952)*
Other ethnicity	127	0.558 (0.385–0.810)*
**Education level**		
Low (illiterate)	657	Ref.
Middle (junior high school)	1414	0.672 (0.516–0.875)*
High (senior high school and above)	222	0.559 (0.407–0.768)*

SHS: secondhand smoke. OR: odds ratio. CI: confidence interval.

* Significant at p<0.05.

## DISCUSSION

In this study, we used data from a survey of chronic disease and nutrition among Chinese adults in Inner Mongolia in northern China during 2015, to investigate the prevalence of SHS exposure, knowledge of the harms of smoking and SHS exposure, and sociodemographic factors associated with SHS exposure among women in Inner Mongolia. The results showed that the rate of exposure to SHS was 64.2% among women in Inner Mongolia during 2015. This was higher than that of a national survey (53.4%) in China^2^, while another report found that only 35% of never-smoking women worldwide were exposed to SHS^16^. The rates of SHS exposure reported globally are nearly half of that in our study. Additionally, the rates in developed countries, such as in the United States and Spain^17,18^, are also far lower than in our study. A previous study revealed that rates of exposure to SHS among non-smoking women in Brazil, India, Turkey, and Vietnam, were 20.7%, 37.9%, 51.7%, and 68.8%, respectively^19^. Interestingly, Vietnam’s exposure rate was slightly higher than that in our study. The disparity in SHS exposures is most likely associated with different knowledge levels regarding smoking and the degree of tobacco control measures implemented in each country.

Importantly, our study showed that SHS exposure was most prevalent among young women in Inner Mongolia (69.0%). Additionally, multivariate logistic regression showed that young women had the highest risk of exposure to SHS among all women. In the present study, younger women were more likely to be exposed to SHS, consistent with the findings of other studies. Surveillance in Thailand showed that young women tended to be exposed to and were most affected by SHS^20^. Similar findings have also been reported in Bangladesh^21^. One explanation could be related to the concept of respect for older people in China, where young women have more societal restrictions on their autonomy and are discouraged from not allowing others around them to smoke, which increases their risk of SHS exposure. Young women aged 12–20 years are vulnerable to SHS exposure, and women are most often affected by their family members^22^. Although several studies have shown that tobacco use among adult women is relatively low in developing countries, the trend in tobacco use has risen rapidly among young women, which is probably related to the increased risk of SHS exposure among never-smoking women^23^. The rate of smoking among young women under the age of 40 years in China increased from 2003 to 2013, and more younger women started to smoke^24^. Young women are not only affected by SHS from men but also from their female peers, which may be related to the highest SHS exposure prevalence found among young women. Recently, the market share of slim cigarettes has also risen sharply. Because such cigarettes were originally designed for women, this suggests that young women might be the new target population for tobacco companies and that increasingly more women have been induced to start smoking and at younger ages. Policies of public health promotion in China should not only encourage men to quit smoking but should also protect women, especially young women, from SHS exposure.

Of particular concern in our results was the frequency of exposure to SHS among young women, with 49.9% of young women exposed to SHS every day. A literature review revealed that women are regularly exposed to SHS in their daily life, for an average 5.5 hours per day^25^. Because SHS is ubiquitous in many environments, a lack of awareness about the negative effects of SHS exposure is common among women. Poor knowledge about the effects of smoking, SHS exposure, and incorrect yet widely held notions that low-tar cigarettes have lower risks than regular cigarettes are common^26^. Our results also showed that women had low levels of knowledge about smoking and SHS exposure, and lacked correct understanding about low-tar cigarettes. The importance of educating the public about the dangers of SHS has been given scant attention. A previous study in China suggested that knowledge regarding the risk of smoking and SHS exposure was poorer in women than in men^27^. Owing to this poor knowledge about the disease-causing effects of smoking and SHS, many women have not yet understood that exposure to SHS presents a potential risk factor; therefore, many women are not motivated to take measures to avoid SHS. Incorrect knowledge about low-tar cigarettes is widespread owing to misinformation promoted by tobacco companies^28^. Tobacco producers have declared that low-tar cigarettes are less harmful than regular cigarettes, which can mislead young women into beginning to smoke. Enhancing understanding among non-smoking women about low-tar cigarettes should be a priority to ultimately improve knowledge levels among young populations regarding the risks of these products.

Young women are exposed to SHS at home and in their workplaces, which might be associated with our finding of the highest risk of SHS exposure among young women. As our results show, most young women were married and employed, and the high risk of exposure to SHS might come from family members and co-workers who smoke. Women are less likely to express disapproval with SHS owing to others’ smoking. A study in Malaysia indicated that a high prevalence of smoking in men would increase the likelihood of SHS exposure among never-smoking women, which suggests that young women at high risk of SHS exposure require greater attention^29^. The home is the predominant source of exposure to SHS because it is where many women spend most of their time^2^. The current situation of SHS exposure among women at home and in the workplace cannot be ignored, and women should be a target population for implementing intervention measures to reduce SHS exposure at home and in public places^30^.

Low education levels were shown to be a risk factor of SHS exposure among never-smoking women. Our results indicated that compared with illiterate women, those with middle or high levels of education (junior high school, senior high school and above) had lower risks of SHS exposure, which is consistent with previous studies^31^. An earlier study in six counties of China also reported that participants with low education levels were more likely to be exposed to SHS^32^. The hazards of SHS exposure may be better understood by well-educated women, which may be related to the risk of negative health outcomes owing to SHS exposure. Women with high levels of education might have correct attitudes toward exposure to SHS in the home or workplace, which would lead to these women being more conscious about avoiding exposure to SHS^33^. Improving awareness about the risks of SHS would contribute to more effective health promotion among women. Additionally, women with high levels of education have non-dismissive attitudes about tobacco control policies and the risk of SHS, which leads to greater compliance with smoking bans in their home or workplace^34^. This is particularly important because smoking bans at home have been found to be effective in limiting family members’ exposure to SHS, especially young people. Moreover, compared with Han women, their counterparts of Mongolian or other ethnicities had a lower risk of SHS exposure. This was probably associated with a lower prevalence of smoking and improved knowledge and correct attitudes regarding the risks of smoking and SHS among women of Mongolian and other ethnicities.

Exposure to SHS has been linked to a number of negative health outcomes for women. Although the Chinese government ratified the Framework Convention on Tobacco Control (FCTC) in 2005, no national smoking ban in public places has been implemented^35^. Mongolia is located in northern China, where the population has low socioeconomic and educational levels. Comprehensive smoke-free policies have also not been fully implemented in public places in this region. A lack of tobacco control bans leads to low levels of awareness about the dangers of SHS exposure among women. Furthermore, young women might not attach much importance to the negative effects of SHS exposure for their health. Smoking at younger ages might be an emerging trend in Inner Mongolia, where most residents begin smoking at an age of 16–24 years, which suggests that youth exposure to SHS in Inner Mongolia requires greater attention^12^. Young women in particular might choose to smoke to lose weight or regulate their mood, and are more likely to ignore the risks of smoking and SHS^36^. This is of particular concern considering the evidence regarding SHS exposure among women and a lack of protection for women against SHS exposure. This situation raises serious concerns about the status of SHS exposure and health among young women in Inner Mongolia.

### Limitations

There were several limitations in our study. SHS exposure was self-reported without cotinine measurement; hence, our results are subject to recall bias. This was a cross-sectional study, which limits inferences about causal relationships. Additionally, we did not identify sources of vulnerability among never-smoking women.

## CONCLUSIONS

This study revealed that the highest prevalence of SHS exposure was among young women in Inner Mongolia during 2015. Exposure to SHS was associated with low levels of education and deficient knowledge regarding SHS as the most important determinants of SHS exposure. Therefore, greater public health attention is needed to increase avoidance of SHS exposure and improve the health status of young women in Inner Mongolia.
