# Supplementary material for: Prevalence, knowledge and education level associated with secondhand smoke exposure among never-smoking women in Inner Mongolia, Northern China
Source: Tob Induc Dis. 2020 Apr 24;18:35. doi: 10.18332/tid/119162 (PMC7199659; doi:10.18332/tid/119162)
Supplement: Supplementary file 1 [file TID-18-35-s1.pdf]

Table S1. Characteristics of never-smoking women in Inner Mongolia, 2015, by exposure to secondhand smoke (SHS) (N=2293)

Variables	Female		$\chi 2$	*P*-value
	No exposure to SHS	Exposure to SHS		
**Age**			34.283	< 0.001**
Elderly women	257(45.89)	303(54.11)		
Middle-aged women	342(33.53)	678(66.47)		
Young women	221(31.00)	492(69.00)		
**Ethnicity**			16.259	< 0.001**
Han	639(34.23)	1228(65.77)		
Mongolian	116(38.80)	183(61.20)		
Other minority	65(51.18)	62(48.82)		
**Marital status**			20.569	< 0.001**
Single	18(35.29)	33(64.71)		
Married	731(34.63)	1380(65.37)		
Widowed/Divorced	71(54.20)	60(45.80)		
**Education**			2.793	0.247
Low(Illiteracy)	234(35.62)	423(64.38)		
Middle(Junior school)	484(34.23)	930(65.77)		
High(Senior high school)	102(45.95)	120(54.05)		
**Occupation**			11.475	0.003*
Unemployed	234(35.62)	423(64.38)		
Employed	484(34.23)	930(65.77)		
Retired	102(45.95)	120(54.05)		
**Area**			0.001	0.970
Urban	377(35.80)	676(64.20)		
Rural	443(35.73)	797(64.27)		

SHS: secondhand smoke.

**Significant at $p < 0.001$; *Significant at $p < 0.05$.

Table S2. Knowledge about low-tar cigarettes among never-smoking women in Inner Mongolia, 2015 (N=2293)

Variables	Female			$\chi 2$	*P*-value
	Young women	Middle-aged women	Elderly women		
**Did you agree that the harm of low-tar cigarettes is less than regular cigarette**				37.646	＜0.001**
Agreement	256(35.90)	308(30.20)	144(25.71)		
Disagreement	115(16.13)	111(10.88)	59(10.54)		
Unknown	342(47.97)	601(58.92)	357(63.75)		

**Significant at $p < 0.001$.

Table S3. Knowledge about low-tar cigarettes among young never-smoking women in Inner Mongolia, 2015, by exposure to SHS (N=713)

Variables	No exposure to SHS	Exposure to SHS	χ 2	*P*-value
**Did you agree that the harm of low-tar cigarettes is lower than regular cigarette**			6.438	0.040*
Agreement	66(29.86)	190(38.62)		
Disagreement	34(15.38)	81(16.46)		
Unknown	121(54.75)	221(44.92)		

SHS: secondhand smoke.

*Significant at $p < 0.05$.

© 2020 Nan X. et al.
